# Seryl-tRNA synthetase is involved in methionine stimulation of *β*-casein synthesis in bovine mammary epithelial cells

**DOI:** 10.1017/S0007114519002885

**Published:** 2019-11-12

**Authors:** Wenting Dai, Fengqi Zhao, Jianxin Liu, Hongyun Liu

**Affiliations:** 1 College of Animal Sciences, Zhejiang University, Hangzhou 310058, People’s Republic of China; 2 Department of Animal and Veterinary Sciences, University of Vermont, Burlington, VT 05405, USA

**Keywords:** Bovine mammary epithelial cells, *β*-Casein, Methionine, Mammalian target of rapamycin–general control nonderepressible 2 pathways, Seryl-tRNA synthetase

## Abstract

Despite the well-characterised mechanisms of amino acids (AA) regulation of milk protein synthesis in mammary glands (MG), the underlying specific AA regulatory machinery in bovine MG remains further elucidated. As methionine (Met) is one of the most important essential and limiting AA for dairy cows, it is crucial to expand how Met exerts its regulatory effects on dairy milk protein synthesis. Our previous work detected the potential regulatory role of seryl-tRNA synthetase (SARS) in essential AA (EAA)-stimulated bovine casein synthesis. Here, we investigated whether and how SARS participates in Met stimulation of casein production in bovine mammary epithelial cells (BMEC). With or without RNA interference against SARS, BMEC were treated with the medium in the absence (containing all other EAA and devoid of Met alone)/presence (containing 0·6 mm of Met in the medium devoid of Met alone) of Met. The protein abundance of *β*-casein and members of the mammalian target of rapamycin (mTOR) and general control nonderepressible 2 (GCN2) pathways was determined by immunoblot assay after 6 h treatment, the cell viability and cell cycle progression were determined by cell counting and propidium iodide-staining assay after 24 h treatment, and protein turnover was determined by l-[ring-^3^H_5_]phenylalanine isotope tracing assay after 48 h treatment. In the absence of Met, there was a general reduction in cell viability, total protein synthesis and *β*-casein production; in contrast, total protein degradation was enhanced. SARS knockdown strengthened these changes. Finally, SARS may work to promote Met-stimulated *β*-casein synthesis via affecting mTOR and GCN2 routes in BMEC.

Dairy milk protein is a high-quality protein source for human consumption. To improve the yield and quality of milk protein, dairy researchers have focused on nutrient regulation of milk protein production in the past decades. Among the nutritional strategies, amino acids (AA) have emerged as important signalling molecules in regulating milk protein synthesis beyond their basic role as building blocks for proteins^(1–6)^. In addition, AA play important roles in the regulation of cell growth^(7)^ and protein metabolism^(8,9)^. As a limiting and essential amino acid (EAA), methionine (Met) exerted remarkable regulatory effects on milk protein production in dairy cows *in vivo* ^(10,11)^ and in mammary epithelial cells *in vitro* ^(12,13)^.

Once Met is transported and absorbed into mammary epithelial cell, its effects on milk protein production are generally regulated by two AA-sensing kinases: mammalian target of rapamycin (mTOR) complex and general control nonderepressible 2 (GCN2)^(14)^. Importantly, mTOR and GCN2 orchestrate cellular adaptations to AA status together and balance intracellular protein metabolism in BMEC: under Met sufficiency, the mTOR system is activated and subsequently stimulates the translation initiation and protein synthesis^(5,15)^; on the contrary, Met limitation predominantly activates the GCN2 pathway, which subsequently prevents global translation and arrests cell growth^(16)^. Although the roles of mTOR and GCN2 pathways in regulating milk protein synthesis were identified in the mammary gland (MG) of humans and mice^(6,17–19)^, the molecular mechanisms underlying Met-mTOR and Met-GCN2 signalling cascades in bovine mammary epithelial cell (BMEC) remain further clarified.

In the past decade, new roles of aminoacyl-tRNA synthetase (AARS) have been identified beyond translation, including regulation of transcription and translation, RNA splicing, immune responses and cellular homeostasis^(20)^. Apart from sestrin2 as a leucine sensor^(21)^, leucyl-tRNA synthetase has been reported to sense leucine availability and initiate molecular events leading to mTOR activation in human 293T cells^(22)^. Moreover, Wang *et al.* uncovered a role of leucyl-tRNA synthetase in the promotion of mammary casein production through activating mTOR pathway in primary BMEC and MG explants^(23)^. Recent studies in our laboratory identified a differential expression of seryl-tRNA synthetase (SARS) in the lactating MG of dairy cows fed low-quality *v*. high-quality forages^(24,25)^ and during different stages of lactation^(26,27)^. These observations have implicated a potential regulatory role of SARS in AA-stimulated milk protein synthesis in the MG.

The objective of the present study was to investigate whether and how SARS is involved in the regulation of Met-stimulated casein synthesis in primary BMEC. We examined the effects of Met (deficiency/sufficiency) and SARS knockdown on cell viability, protein turnover, casein synthesis and expression of proteins involved in mTOR and GCN2 pathways in BMEC. Results suggested that SARS is involved in Met-stimulated *β*-casein synthesis mainly through activating mTOR pathway and inhibiting GCN2 pathway in BMEC.

## Methods

### Materials

Fetal bovine serum (10099141), DMEM-F12 (1133005) and penicillin-streptomycin (10378016) were purchased from Gibco. Trypsin/EDTA (9002-07-7) was purchased from Sigma-Aldrich. Dulbecco’s modified Eagle’s medium (DMEM) devoid of Met alone or all EAA was both custom-made from Gibco. l-Glutamine (G8540), l-Met (M9625), insulin (from bovine pancreas, I5500), prolactin (from sheep pituitary, 9002-62-4), hydrocortisone (H0888), transferrin (from bovine, T1283) and epidermal growth factor (from murine submaxillary gland; E4127) were purchased from Sigma-Aldrich. l-[ring*-* ^3^H_5_]phenylalanine (Phe) (NET1122001MC) was obtained from PerkinElmer Inc. The sources of all the antibodies used are presented in online Supplementary Table S1.

### Ethics statement

The primary BMEC were used in the present study, and the Institutional Animal Care and Use Committee of Zhejiang University of China approved the care and handling of the dairy cows from which the mammary cells were obtained.

### Cell culture

The BMEC were isolated as previously described^(28)^. Briefly, MG tissues were obtained from three healthy mid-lactation dairy cows (age 58 (sd 7) months; days in milk 92–118) and cut into 1 mm^3^ pieces with sterile surgical scissors. The pieces were washed with PBS buffer several times and incubated in DMEM-F12 with 5 μg/ml transferrin, 5 μg/ml insulin, 5 μg/ml prolactin, 1 μg/ml hydrocortisone, 10 ng/ml epidermal growth factor, 1 % glutamine, 1 % penicillin-streptomycin and 10 % fetal bovine serum at 37°C in a humidified atmosphere containing 5 % CO_2_. After the cells migrated from tissues and covered up to 80 % of the flask bottom surface, the tissues were removed. BMEC were separated from fibroblasts using their different sensitivities to trypsin digestion. Approximately 1 × 10^6^ cells were cultured in 10 cm^2^ plastic dishes in the above DMEM-F12 complete medium. Cells were incubated at 37°C in a humidified atmosphere containing 5 % CO_2_.

### Experimental design and treatments

**Fig. 1.**
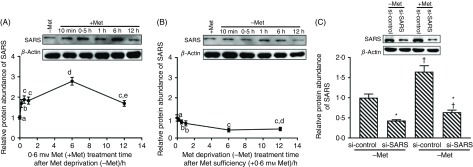
Methionine (Met) stimulates seryl-tRNA synthetase (SARS) expression in bovine mammary epithelial cells (BMEC). The relative protein abundance of SARS in BMEC first treated with Met-deprived medium (–Met) for 12 h and then re-supplemented with 0·6 mm Met medium (+Met) for 10 min, 0·5, 1, 6 or 12 h (A); first treated with 0·6 mm Met medium (+Met) for 12 h and then deprived of Met (–Met) for 10 min, 0·5, 1, 6 or 12 h (B). (C) The relative protein abundance of SARS in BMEC transfected with si-SARS and treated with Dulbecco’s modified Eagle’s medium (DMEM) in the absence and presence of Met (−/+Met, *P*_Met_ < 0·001, *P*_SARS_ < 0·001, *P*_interaction_ < 0·001). –Met, DMEM without Met; +Met, Met-deprived medium supplemented with 0·6 mm Met; si-control, scramble small interfering RNA (siRNA); si-SARS, specific siRNA to SARS. In all panels, data represent mean values with their standard errors of three independent experiments, and at least three wells per treatment within each independent experiment. The relative protein abundance of SARS in (A) and (B) was analysed using one-way ANOVA followed by Bonferroni adjustment; ^a,b,c,d,e^ unlike letters indicate significant differences (*P* < 0·05). The relative protein abundance of SARS in (C) was analysed using two-way ANOVA followed by Bonferroni adjustment, and * *P* < 0·05 *v.* si-control and †*P* < 0·05 *v.* –Met.

The previous study^(29)^ and the supplementary materials in the present study showed that Met supplementation at the concentration of 0·6 mm enhanced the BMEC viability and *β*-casein synthesis at the highest levels. Therefore, in the present study, we selected 0·6 mm Met to treat the BMEC. Totally, 3 × 10^5^ BMEC were seeded in six-well plates and grew to approximately 80–90 % confluence. Cells were serum-starved overnight and then treated with a Met-deprived medium (DMEM with other EAA and devoid of Met alone, −Met) over 12 h followed by a Met re-supplementation medium (DMEM devoid of Met and then supplied with 0·6 mm Met, +Met) for 10 min, 0·5, 1, 6 and 12 h. Likewise, the cells were first treated with Met supplementation medium followed by Met-deprived medium. After treatments, cells were immediately collected and lysed using 0·2 ml of lysis buffer for 2 h on ice as previously described^(30)^. The cell homogenates were centrifuged at 12 000 ***g*** for 5 min at 4°C, and the supernatants were collected as protein samples for the Western blot analysis. To measure the effects of SARS or methionyl-tRNA synthetase (MARS) knockdown on Met-stimulated casein synthesis, cells were first transfected with SARS or MARS small interfering RNA (siRNA) (si-SARS or si-MARS) overnight followed by 12 h serum starvation and then treated with the Met deprivation or supplementation medium for 6 h to collect RNA and protein samples for PCR and Western blot analysis, 24 h for cell viability and cell cycle assay and 2 d for protein turnover assay. Here in order to acquire the prominent effects of each measured item, we selected the various lengths of incubation times of BMEC. Because, our previous study^(31)^ and this work in the following Fig. 1 found that 6 h treatment of EAA/Met deprivation and sufficiency in BMEC both demonstrated the most prominent effects on the protein expression and of SARS and casein. Therefore, we chose 6 h treatment of Met deprivation and sufficiency to determine their effects on the related mRNA and protein alterations. Actually, in our preliminary experiments, BMEC were treated with Met deprivation/sufficiency combined with SARS knockdown for varying time points (6, 12, 24, and 48 h; data not shown), and we found that the changes of cell viability among the four groups were the most remarkable at 24 h treatment. In the present study, we presented the data of cell viability after 24 h treatments. As protein turnover undergoes a dynamic process, the cellular balance between protein synthesis and degradation needs two and more days^(32)^. Based on our previous studies^(31,33)^, we usually treated bovine MG for 2 d to determine their protein turnover rates. Taking consideration of the theoretical and practical knowledge, here we chose 2 d for protein turnover assay.

### RNA interface transfection

The specific siRNA (online Supplementary Table S2) used in the present study and the negative control siRNA were synthesised by GenePharma. Before transfection, 3 × 10^5^ cells per well were seeded in a six-well plate. On the following day, cells were transfected with 80 nm si-SARS and si-MARS using a Lipofectamine™ RNAiMAX transfection reagent (Invitrogen) in lactogenic medium without antibiotics according to the manufacturer’s instructions.

### Protein turnover assay

The protein turnover was determined by the isotope labelling method. To measure protein synthesis, 3 × 10^5^ cells per well seeded in six-well plate were first transfected with si-SARS overnight followed by 12 h serum starvation and then treated with DMEM containing 5 % fetal bovine serum in the absence and presence of 0·6 mm Met for 2 d. The cells were then washed three times with 2 ml of DMEM (devoid of Met) and then treated for an additional 24 h in 2 ml of DMEM containing 1 mm l-Phe + 0·8 μCi l-[ring-2,4-^3^H_5_]Phe (specific radioactivity = 500 dpm/nm) and in the absence/presence of 0·6 mm Met. For measuring the rate of protein degradation, BMEC were pulse-labelled with l-[ring-2,4-^3^H_5_]Phe in a short time (3 h) and the decay of the labelled protein while chasing with unlabelled precursor was monitored. Briefly, cells were cultured for 24 h in 2 ml of DMEM containing 0·1 mm l-Phe + l-[ring-2,4-^3^H_5_]Phe and in the absence/presence of 0·6 mm Met. Subsequently, cells were washed three times with 2 ml of DMEM devoid of Met to deplete extracellular free l-[ring-2,4-^3^H_5_]Phe and then cultured for 3 h in 2 ml of the Met-deprived/supplemented DMEM containing 1 mm l-Phe. The radioactivities of l-[ring-2,4-^3^H_5_]Phe in the intracellular pool and in the medium were measured by liquid-scintillation spectrometry. The rate of protein degradation was calculated by dividing the amount of l-[ring-2,4-^3^H_5_]Phe radioactivity released to the incubation medium during the final 3 h incubation period by the specific radioactivity of protein bound l-[ring-2,4-^3^H_5_]Phe^(34,35)^.

### Cell viability assay

The cell viability assay was measured using a cell counting kit-8 (Beyotime). Approximately 3 × 10^5^ cells per well seeded in a six-well plate were first transfected with si-SARS overnight and then were set up at 1 × 10^4^ cells/well in a ninety-six-well plate. Prior to treatments, cells were 12 h serum-starved and then treated with the medium in the presence and absence of 0·6 mm Met for 24 h. After the treatment, 20 μl/well cell counting kit-8 solution was added and cells were incubated for another 2 h. The viable cells were measured by spectrophotometric absorbance at 450 nm with a microplate reader (Spectramax M5).

### Cell cycle assay

The proportion of BMEC in different stages in cell cycle was determined by propidium iodide-staining and flow cytometry assay. First, cells seeded at 3 × 10^5^ cells/well in six-well plates were transfected with si-SARS overnight followed by 12 h serum starvation. Cells were then treated with the medium in the presence and absence of 0·6 mm Met for 24 h. Subsequently, cells were washed three times with ice-cold PBS, fixed overnight with ice-cold 70 % ethanol (v/v) in water at 4°C, washed three times with PBS and resuspended with PBS containing 0·1 mg/ml RNase A and 5 μg/ml propidium iodide for 0·5 h at 37°C in dark. After three additional washes with PBS, cells were resuspended in PBS and analysed using a FACS Calibur (BDFACS Aria^TM^ Cell Sorter; Becton-Dickinson). The colour intensity of propidium iodide-stained BMEC in different cell cycle phases was determined, and the resulting data were analysed using Modfit software (version 3.0; Verity Software House).

### Quantitative reverse transcription PCR assay

Total RNA was extracted from mammary cells with an Aidlab Rneasy kit (Aidlab) and reverse-transcribed for cDNA synthesis using a PrimeScriptRT Reagent Kit with gDNA Eraser (Takara). The quantitative reverse transcription PCR was performed in triplicate (three reactions per RNA sample) using the Applied Biosystems 7500 real-time PCR system (Applied Biosystems). The 20 μl reaction included 50 ng of reverse transcription product, 40 nm of each forward and reverse primers (online Supplementary Table S3, designed by Primer 5 software (Premier Biosoft International)) and the SYBR Premix Taq (Takara). The running programme was one cycle of 95°C for 30 s plus forty cycles of amplification at 95°C for 5 s and 60°C for 34 s, followed by the melt curve of an additional 15 s at 95°C, 1 min at 60°C and 15 s at 95°C. The relative mRNA abundance of target genes was normalised to the expression levels of ribosome protein 9 and *β*-actin and calculated by the 2^−ΔΔCt^ method^(36)^.

### Western blot analysis

The protein concentrations of the cell lysate were determined using the Bradford protein assay kit using the bovine serum albumin as the protein standard (Beyotime). Equal amounts of protein (25 μg) per sample were separated on 10 % SDS polyacrylamide gels and then transferred onto 0·45 μm PVDF membranes (Millipore). The membranes were blocked with a blocking buffer (Beyotime) at room temperature for 2 h, followed by 4 h incubation at room temperature with the primary antibodies mentioned in online Supplementary Table S1. After washing with (TRIS-buffered saline containing 0·02 % (v/v) Tween-20 (TBST) three times, the membranes were then incubated with secondary goat-anti-rabbit IgG (Beyotime, dilution 1:500) or goat-anti-mouse IgG (Beyotime, dilution 1:500) conjugated with horseradish peroxidase in a blocking buffer for 4 h at 37°C. The membranes were then washed with TBST three times and immediately incubated with enhanced chemiluminescence Western blotting substrate kits (Beyotime), followed by visualisation using a chemiluminescence imaging system (CLiNX Science Instrument). The relative intensities of the bands were calculated with ImagePro Plus 6.0 software (Media Cybernetics). The *β*-actin protein was used as the reference protein.

### Statistical analyses

Each experiment was conducted with three replicates and independently repeated three times with the use of cells isolated from different animals (*n* 3). Data are presented as mean values with their standard errors. The relative protein abundance of SARS under Met deprivation/sufficiency at different treatment time points was analysed using the one-way ANOVA. The multiple means were compared using Bonferroni adjustment. Two-way ANOVA followed by Bonferroni correction was used to test the effects of Met availability and SARS knockdown on cell viability, cell cycle and the relative protein abundance of the proteins (SARS, *β*-casein, the mTOR and GCN2 pathway-related proteins). All *P* values < 0·05 were considered statistically significant. All statistical analyses were performed with SAS software (version 9.2; SAS Institute).

## Results

### Methionine stimulates seryl-tRNA synthetase expression in bovine mammary epithelial cells

To investigate the effects of individual EAA on the protein abundance of SARS, BMEC were treated with DMEM containing all EAA, no EAA or ten individual EAA alone. As shown in online Supplementary Fig. S1A, the protein abundance of SARS was similar in cells treated with Met, Phe and isoleucine alone or with all EAA, but higher than the cells treated with other eight individual EAA (*P* < 0·05). Furthermore, Met stimulated SARS protein expression in a dose-dependent manner. When Met concentration increased from 0·1 to 0·6 mm, the protein abundance of SARS was gradually enhanced (*P* < 0·05) and reached the highest level (online Supplementary Fig. S1B). In addition, compared with the Met deprivation group, supplementation with 0·6 mm Met increased the relative mRNA abundance of SARS, MARS and cysteinyl-tRNA synthetase (*P* < 0·05), among which the mRNA abundance of SARS was the highest (*P* < 0·05; online Supplementary Fig. S1C).

To further examine the stimulating effects of Met on SARS protein expression, BMEC were first deprived of Met for 12 h and then re-supplemented with 0·6 mm Met. At 6 h of Met re-supplementation, the relative protein abundance of SARS reached the highest among other time points (*P* < 0·05; Fig. 1(A)). In contrast, when BMEC were first treated with 0·6 mm Met medium for 12 h followed by Met-deprived medium, the changes in the SARS protein abundance showed the opposite trends (*P* < 0·05; Fig. 1(B)). Besides, the knockdown of SARS by siRNA decreased SARS protein abundance by 73 and 84 % in Met-deprived/supplemented groups, respectively (*P* < 0·05; Fig. 1(C)). There existed an interaction between Met availability and SARS knockdown on the protein expression of SARS in BMEC.

### Seryl-tRNA synthetase regulates the effects of methionine on cell viability and cell cycle in bovine mammary epithelial cells

**Fig. 2.**
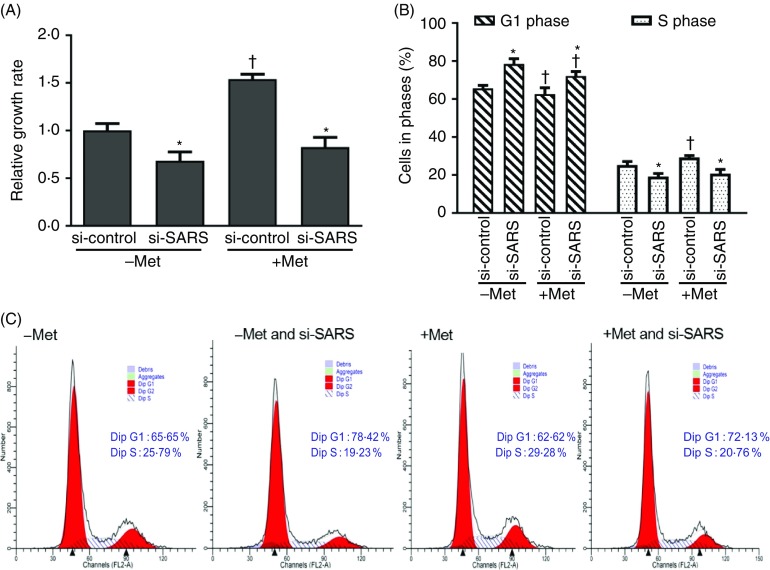
Roles of seryl-tRNA synthetase (SARS) in the effects of methionine (Met) on cell viability and cell cycle in bovine mammary epithelial cells (BMEC). The cell viability ((A) *P*_Met_ < 0·001, *P*_SARS_ < 0·001, *P*_interaction_ = 0·0101) and the percentages of cells in different cell phases compared with the total cells in BMEC ((B) and (C) G1 phase, *P*_Met_ < 0·001, *P*_SARS_ = 0·0131, *P*_interaction_ < 0·001; S phase, *P*_Met_ = 0·003, *P*_SARS_ = 0·0213, *P*_interaction_ < 0·001 in BMEC transfected with si-SARS and treated with Dulbecco’s modified Eagle’s medium (DMEM) in the absence and presence of Met (−/+Met)). –Met, DMEM without Met; +Met, Met-deprived medium supplemented with 0·6 mm Met; si-control, scramble small interfering RNA (siRNA); si-SARS, specific siRNA to SARS. In all panels, data represent mean values with their standard errors of three independent experiments. All data were analysed using two-way ANOVA followed by Bonferroni adjustment, and * *P* < 0·05 *v.* si-control and † *P* < 0·05 *v.* –Met.

To elucidate the function of SARS in the mammary cellular processes, we examined its role in cell viability and cell cycle of BMEC in response to Met. As shown in Fig. 2(A), the relative cell viability was repressed in the Met-deprived group compared with Met-supplemented group (*P*_Met_ < 0·001) and this reduction in mammary cell viability was also reduced by SARS knockdown (*P*_SARS_ < 0·001). Besides, there existed an interaction between Met availability and SARS knockdown on bovine mammary cell viability. As shown in Fig. 2(B) and (C), the percentages of mammary cells in S phase were increased by Met supplementation (S phase, *P*_Met_ = 0·003). In contrast, SARS knockdown by RNA interference increased mammary cell percentages in G1 phase (*P*_SARS_ = 0·0131) but decreased mammary cell percentages in S phase (*P*_SARS_ = 0·0213). Besides, there also existed an interaction between Met availability and SARS knockdown in regulation of mammary cell cycle progression in BMEC (*P*_interaction_ < 0·05).

### Seryl-tRNA synthetase mediates the effects of methionine on protein turnover and *β*-casein synthesis in bovine mammary epithelial cells

**Fig. 3.**
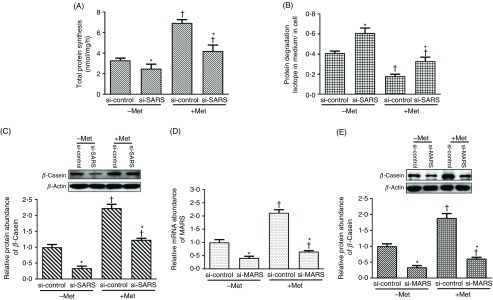
Roles of seryl-tRNA synthetase (SARS) in methionine (Met)-involved protein metabolism in bovine mammary epithelial cells (BMEC). The total protein synthesis ((A) *P*_Met_ < 0·001, *P*_SARS_ < 0·001, *P*_interaction_ < 0·001), protein degradation ((B) *P*_Met_ < 0·001, *P*_SARS_ < 0·001, *P*_interaction_ < 0·001) and *β*-casein ((C) *P*_Met_ < 0·001, *P*_SARS_ < 0·001, *P*_interaction_ = 0·0152) in BMEC transfected with si-SARS and then treated with Dulbecco’s modified Eagle’s medium (DMEM) in the absence and presence of Met (−/+Met). The relative mRNA abundance of MARS ((D) *P*_Met_ < 0·001, *P*_MARS_ < 0·001, *P*_interaction_ < 0·001) and relative protein abundance of *β*-casein in BMEC ((E) *P*_Met_ < 0·001, *P*_MARS_ < 0·001, *P*_interaction_ < 0·001) in BMEC transfected with si-MARS (methionyl-tRNA synthetase) and then treated with DMEM in the absence and presence of Met (−/+Met). –Met, DMEM without Met; +Met, Met-deprived medium supplemented with 0·6 mm Met; si-control, scramble small interfering RNA (siRNA); si-SARS/si-MARS, specific siRNA to SARS/MARS. In all panels, data represent mean values with their standard errors of three independent experiments. All data were analysed using two-way ANOVA followed by Bonferroni adjustment, and * *P* < 0·05 *v.* si-control and † *P* < 0·05 *v.* –Met.

To explore whether SARS is involved in Met-stimulated protein metabolism, we studied its effects on protein turnover in BMEC in response to Met. Our data showed that when Met was deprived or SARS expression was knocked down, the total protein synthesis rates were reduced (Fig. 3(A), *P*_Met_ < 0·001, *P*_SARS_ < 0·001), but the rates of protein degradation were enhanced (Fig. 3(B), *P*_Met_ < 0·001, *P*_SARS_ < 0·001). Besides, Met deprivation and SARS knockdown both depressed the protein abundance of *β*-casein (Fig. 3(C), *P*_Met_ < 0·001, *P*_SARS_ < 0·001). These two factors (Met availability and SARS knockdown) existed an overall interaction effects on protein metabolism and the *β*-casein synthesis in BMEC (*P*_interaction_ < 0·05).

To explore the specificity of different tRNA, the effect of MARS knockdown on the protein abundance of *β*-casein was examined. The relative mRNA abundance of MARS was severely depressed by MARS siRNA regardless of Met presence (*P*_MARS_ < 0·05; Fig. 3(D)), and the protein abundance of *β*-casein was also reduced by MARS knockdown (*P*_MARS_ < 0·001; Fig. 3(E)). There also existed an interaction effect between Met and MARS knockdown on the *β*-casein expression in BMEC (*P*_interaction_ < 0·001).

### Involvement of seryl-tRNA synthetase in the activation of mammalian target of rapamycin and repression of general control nonderepressible 2 pathways in bovine mammary epithelial cells in response to methionine

**Fig. 4.**
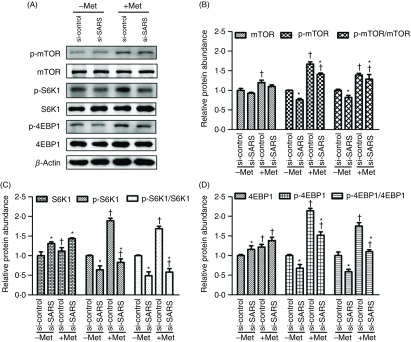
Involvement of seryl-tRNA synthetase (SARS) in activation of the mammalian target of rapamycin (mTOR) signalling pathway in bovine mammary epithelial cells (BMEC) in response to methionine (Met). (A) Representative Western blots of mTOR signalling molecules in BMEC transfected with si-SARS and then treated with Dulbecco’s modified Eagle’s medium (DMEM) in the absence and presence of Met (−/+Met). The relative protein abundance of mTOR (*P*_Met_ = 0·015, *P*_SARS_ = 0·1627, *P*_interaction_ = 0·0778), phosphorylated (p-) mTOR (*P*_Met_ < 0·001, *P*_SARS_ < 0·001, *P*_interaction_ = 0·0125) and the rate of p-mTOR to total mTOR (*P*_Met_ < 0·001, *P*_SARS_ < 0·001, *P*_interaction_ < 0·001) (B); S6K1 (*P*_Met_ < 0·001, *P*_SARS_ = 0·0241, *P*_interaction_ = 0·3898), p-S6K1 (*P*_Met_ < 0·001, *P*_SARS_ < 0·001, *P*_interaction_ < 0·001) and the rate of p-S6K1 to total S6K1 (*P*_Met_ < 0·001, *P*_SARS_ < 0·001, *P*_interaction_ = 0·4778) (C); 4EBP1 (*P*_Met_ < 0·001, *P*_SARS_ = 0·041, *P*_interaction_ = 0·0264), p-4EBP1 (*P*_Met_ < 0·001, *P*_SARS_ < 0·001, *P*_interaction_ = 0·0316) and the rate of p-4EBP1 to total 4EBP1 (*P*_Met_ < 0·001, *P*_SARS_ < 0·001, *P*_interaction_ = 0·0312) (D) in BMEC transfected with si-SARS and then treated with DMEM in the absence and presence of Met (−/+Met). –Met, DMEM without Met; +Met, Met-deprived medium supplemented with 0·6 mm Met; si-control, scramble small interfering RNA (siRNA); si-SARS, specific siRNA to SARS. In all panels, data represent mean values with their standard errors of three independent experiments. All data were analysed using two-way ANOVA followed by Bonferroni adjustment, and * *P* < 0·05 *v.* si-control and † *P* < 0·05 *v.* –Met.

To better understand how SARS exerts its effects on casein production of BMEC in response to Met, we studied whether Met could affect two major pathways (mTOR and GCN2) that regulate milk protein synthesis and whether there exists any possible involvement of SARS in these effects. As shown in Fig. 4, Met supplementation enhanced the protein abundance of phosphorylated mTOR, S6K1 and 4EBP1 (*P*_Met_ < 0·05, Fig. 4(B)–(D)) and the ratios of phosphorylated protein to total protein of these three proteins (*P*_Met_ < 0·05, Fig. 4(B)–(D)) compared with the Met-deprived groups. Furthermore, the phosphorylation ratios of the mTOR signalling molecules were enhanced in BMEC without SARS knockdown (*P*_SARS_ < 0·05, Fig. 4). Besides, Met availability and SARS knockdown have a general interactive effect on the activation of mTOR pathway (p-mTOR, *P*_interaction_ = 0·0125; p-mTOR to mTOR, *P*_interaction_ < 0·001; p-S6K1, *P*_interaction_ < 0·001; p-4EBP1, *P*_interaction_ = 0·0316; p-4EBP1 to 4EBP1, *P*_interaction_ = 0·0312). In contrast, no significant interaction on total mTOR, S6K1 and 4EBP1 protein expression existed between Met availability and SARS knockdown. These results indicated that SARS may be involved in the activation of the mTOR pathway in BMEC stimulated by Met.

**Fig. 5.**
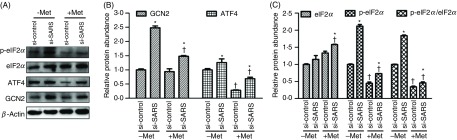
Involvement of seryl-tRNA synthetase (SARS) in methionine (Met) inhibition of the GCN2 signalling pathway in bovine mammary epithelial cells (BMEC). (A) Representative Western blots of the relative protein abundance of ATF4, GCN2, eIF2*α* and phosphorylated (p-) eIF2*α* in the BMEC transfected with si-SARS and then treated with Dulbecco’s modified Eagle’s medium (DMEM) in the absence and presence of Met (−/+Met). –Met, DMEM without Met; +Met, Met-deprived medium supplemented with 0·6 mm Met. The relative protein abundance of GCN2 (*P*_Met_ < 0·001, *P*_SARS_ < 0·001, *P*_interaction_ = 0·5839) and ATF4 (*P*_Met_ < 0·001, *P*_SARS_ < 0·001, *P*_interaction_ = 0·0021) (B); eIF2*α* (*P*_Met_ = 0·0346, *P*_SARS_ < 0·001, *P*_interaction_ = 0·0357), p-eIF2*α* (*P*_Met_ < 0·001, *P*_SARS_ < 0·001, *P*_interaction_ < 0·001) and the ratio of p-eIF2*α* to total protein eIF2*α* (*P*_Met_ < 0·001, *P*_SARS_ < 0·001, *P*_interaction_ < 0·001) (C) in BMEC transfected with si-SARS and then treated with DMEM in the absence and presence of Met (−/+Met). –Met, DMEM without Met; +Met, Met-deprived medium supplemented with 0·6 mm Met; si-control, scramble small interfering RNA (siRNA); si-SARS, specific siRNA to SARS. In all panels, data represent mean values with their standard errors of three independent experiments. All data were analysed using two-way ANOVA followed by Bonferroni adjustment, and * *P* < 0·05 *v.* si-control and † *P* < 0·05 *v.* –Met.

Both the presence of Met and SARS knockdown enhanced the protein abundance of ATF4, GCN2 and phosphorylated eIF2*α* (*P*_SARS_ < 0·001, Fig. 5). In contrast, Met supplementation had no effects on the total protein abundance of GCN2 (Fig. 5). Both Met supplementation and SARS knockdown increased the protein abundance of total eIF2*α* (*P*_Met_ = 0·0346; *P*_SARS_ < 0·001; Fig. 5). There existed an interactive effect of the two factors (Met availability and SARS) on the GCN2 signalling molecules (ATF4 and phosphorylated eIF2*α*; *P*_interaction_ < 0·05) except GCN2 itself (*P*_interaction_ = 0·5839). These observations suggested that SARS displayed an inhibitory action on the GCN2 pathway in BMEC, especially when Met was deprived.

## Discussion

**Fig. 6.**
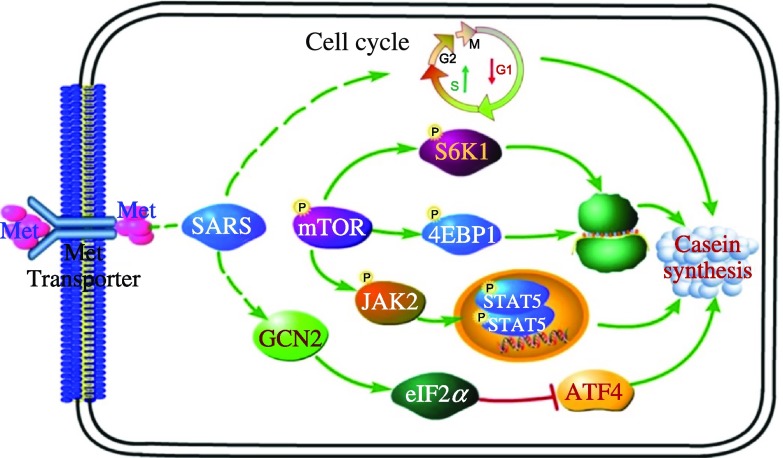
Model illustrating the mechanisms underlying the involvement of seryl-tRNA synthetase (SARS) in methionine stimulation of casein synthesis in bovine mammary epithelial cells (BMEC). The green arrows indicate the promotion effect, and the red bar lines indicate the inhibition effect. The capital letter P indicates the phosphorylation of proteins. mTOR, mammalian target of rapamycin; 4EBP1, eukaryotic translation initiation factor 4E-binding protein 1; S6K1, ribosomal protein S6 kinase *β*-1; GCN2, eukaryotic translation initiation factor 2*α* kinase 4; eIF2*α*, eukaryotic translation initiation factor 2*α*; ATF4, activating transcription factor 4.

Extensive studies demonstrated that AA play a crucial role in the regulation of milk protein synthesis through cell proliferation and the mTOR signalling pathway *in vitro* ^(2,17,18,37,38)^, but the mechanisms how AA regulate milk protein production in BMEC require further studies. Our recent work demonstrated a potential regulatory role of SARS in milk protein production in the MG of dairy cows^(26,27,31)^. In the present study, our data revealed that SARS may be involved in affecting the mTOR and GCN2 routes by Met, thereby regulating milk protein synthesis in BMEC. Among five AARS, the mRNA expression of SARS and MARS was significantly increased by Met supplementation. Thus, the present study establishes that SARS molecule may act as a crucial mediator of Met-stimulated milk protein synthesis in BMEC (Fig. 6).

We first observed Met supplementation promoted cell viability, cell cycle progression and *β*-casein/total protein synthesis, but reduced the protein degradation in BMEC. The results are consistent with most of the previous studies *in vivo* and *in vitro*. Studies reported that the lactation performance (especially the yield of milk and milk protein and milk protein concentration) in dairy cows receiving supplemental dietary Met (25–35 g/d) at early lactation^(39)^ or postruminal infusion of rumen-protected Met (60 g/d) during the peripartal period *in vivo* ^(40)^ was both significantly increased compared with those in groups without Met supplementation. Our study showed that Met supplementation at the concentration of 0·6 mm can significantly promote the casein synthesis and activate mTOR signalling, which were similar to several previous studies^(29,41,42)^.

We found that SARS, MARS and cysteinyl-tRNA synthetase mRNA expressions were significantly stimulated by Met addition. These data suggest that these AARS (especially SARS) might be involved in AA-stimulated milk synthesis. Recent work reported that glycyl-tRNA synthetase regulated the mTOR signalling pathway to promote milk protein and fat synthesis in BMEC^(42,43)^. These observations and our data suggest the crucial regulatory roles of AARS in milk protein synthesis. Indeed, we showed that SARS is involved in Met stimulation of cell viability, cell cycle progression, *β*-casein and total protein synthesis as well as in Met inhibition of protein degradation because SARS knockdown altered the effects of Met. These results were in line with our previous finding that the knockdown of SARS highly reduced cell proliferation, *β*-casein and protein turnover in BMEC without EAA supplementation^(31)^. Additionally, the reduction of *β*-casein under SARS knockdown in the present study was in agreement with the finding of leucyl-tRNA synthetase^(23)^, whose knockdown depressed *β*-casein protein production in BMEC, especially under leucine and Met deprivation. Although the reduction of Met-stimulated *β*-casein synthesis by MARS knockdown was a little more pronounced than the effect of SARS knockdown, the Met-stimulated *β*-casein synthesis was significantly reduced once SARS expression was repressed.

The mTOR signalling pathway is critically involved in AA regulation of milk protein synthesis in the MG^(6,37)^. Once the mTOR system is activated by AA sufficiency, mTOR and its downstream molecules (S6K1 and 4EBP1) are phosphorylated^(44,45)^. In the study, alterations of the protein expression of mTOR-related proteins by Met supplementation were in accordance with previous findings in BMEC supplemented with Met^(29,42)^. However, we found that SARS knockdown depressed mTOR activation under Met stimulation, which is consistent with the inhibition of the mTOR activation by leucyl-tRNA synthetase knockdown in the HEK293T cells^(22)^ and BMEC^(23)^ in response to leucine. In addition, the time dependence of SARS expression stimulated by Met in BMEC may be associated with the mTOR activation. Because the localisation levels of mTOR on the lysosomal outer surface (indication of mTOR activation) were gradually enhanced, when BMEC were first starved by EAA deprivation and then resupplied with EAA for the same time points as the present study^(38)^. The effects corresponding to the increase in EAA treatment time, and at 6 h, the localisation level reached their maximum. Therefore, it appears that the mTOR activation in the present study may be changed depending on the length of treatment time of Met absence or presence, which may lead to this time-dependent SARS synthesis in BMEC. These works suggest that SARS may be responded to mammary cellular Met availability and then regulates *β*-casein production through activating mTOR pathway.

In mammals, GCN2, one of the four eIF2*α* kinases, directly senses the AA deprivation signal by binding to uncharged tRNA, which subsequently is activated by its dimerisation and autophosphorylation^(46)^. The activated GCN2 phosphorylates eIF2*α* to block translation initiation of most mRNA^(47)^. While decreasing global mRNA translation, eIF2*α* phosphorylation drives an increase in the translation of ATF4, which plays a role in initiating the integrated stress response^(48)^. Despite no direct evidence for the association between GCN2 signalling pathway and casein synthesis in ruminants, it has been demonstrated that the activation of the GCN2 pathway would lead to general attenuated global translation and enhanced growth arrest in mice^(16)^. Thus, it is reasonable to assume that the GCN2 activation induced by AA starvation may result in lower milk protein production (including caseins) in the ruminant MG. In the present study, Met deficiency in BMEC enhanced the ATF4 expression and the phosphorylation of eIF2*α*, which were in line with similar alterations in expression patterns of GCN2-related proteins in Met-deficient MEF^(49)^. However, the enhanced expression of GCN2 in the absence of Met was not consistent with the previous finding in MEF in which Met deprivation decreased the GCN2 expression^(49)^. Several factors may account for this difference: the different cell lines used (immortalised MEF *v.* primary BMEC) and the Met starvation time (12 h *v.* 6 h). Although there was not a large change in GCN2 levels in the presence and absence of Met, the inhibitory action of SARS on GCN2 expression was lower in the presence of Met than in the absence of GCN2. Because GCN2 can be specifically activated by ‘uncharged’ tRNA accumulation during amino acid starvation^(50)^, we assume that SARS knockdown directly lowers the Ser-tRNA synthesis and this may be more likely to induce higher ‘uncharged’ tRNA accumulation during Met deprivation, which finally leads to enhanced level of GCN2. In addition, SARS knockdown enhanced the expression levels of the GCN2-related proteins (ATF4 and phosphorylated eIF2*α*) in BMEC and SARS exerted its interaction with Met, suggesting that SARS may be involved in Met-stimulating casein production by inhibiting the GCN2 signalling pathway. Interestingly, Ye *et al.* uncovered an important link between mTOR and GCN2 signalling: the AA deprivation-induced activation of GCN2 up-regulates ATF4 to stimulate the expression of the stress response protein sestrin2^(14)^, which is essential for the repression of mTORC1 by blocking its lysosomal localisation. Thus, we assume that SARS may play a role in linking the functions of the two opposite AA sensing signalling pathways (mTORC1 and GCN2).

The present work elucidates the association of SARS molecule with Met availability in regulation of casein production, which expands the AA regulatory mechanisms in milk protein synthesis in dairy cows and other species. We also showed other AARS, such as MARS and cysteinyl-tRNA synthetase, also have the similar function, but their function mechanisms require further study. Besides, the present study only used RNA interference transfection approach to reduce SARS expression. In addition, future studies should also over-express or knockout SARS gene to deeply explore the role of SARS in regulating milk protein synthesis.

### Conclusions

In summary, in response to Met levels, SARS may function via the following two regulatory mechanisms (Fig. 6): (1) increase the mammary cell population via enhancing cell viability and cell cycle progression; and (2) affect the mTOR and GCN2 routes to promote *β*-casein synthesis. The present study establishes a potential regulatory role of SARS in milk protein synthesis in BMEC and expands our understanding of AA regulation of protein synthesis in the MG.

## Supplementary material

For supplementary material accompanying this paper visit https://doi.org/10.1017/S0007114519002885. click here to view supplementary material
