# Supplementary material for: Seryl-tRNA synthetase is involved in methionine stimulation of β-casein synthesis in bovine mammary epithelial cells
Source: Br J Nutr. 2019 Nov 12;123(5):489–98. doi: 10.1017/S0007114519002885 (PMC7015878; doi:10.1017/S0007114519002885)
Supplement: Supplementary file 1 [file S0007114519002885sup001.docx]

**Supplementary methods**

*Experimental design*

To determine the effects of EAA on the protein expression of SARS, cells were treated with the DMEM medium containing all EAA, no EAA, or 10 individual EAA (0.4 mM arginine, Arg; 0.2 mM histidine, His; 0.8 mM isoleucine, Ile; 0.8 mM leucine, Leu; 0.8 mM lysine, Lys; 0.4 mM phenylalanine, Phe; 0.4 mM tyrosine, Tyr; 0.8 mM valine, Val; 0.2 mM Methionine, Met; 0.8 mM threonine, Thr) with lactogenic hormones for 24 h.

To determine the effects of different concentrations of Met on the protein expression of SARS, cells were treated with the DMEM medium containing 0, 0.1, 0.2, 0.4, 0.3, 0.8, 1.6 or 2.4 mM Met including the lactogenic hormones for 24 h. To determine the effects of Met on the gene expression of different AARS, cells were treated with the DMEM medium containing 0.6 mM Met (+Met) or no Met (–Met) with the hormones for 24 h.

*Statistical analyses*

Each experiment was conducted with 3 replicates, and each experiment was independently repeated 3 times with the cells isolated from different animals (n=3). Data are presented as the mean ± SEM. The relative protein abundances of SARS in cells under different individual EAA and under different Met concentration treatments were both analyzed using the One-way ANOVA. Here, we have made the typical measures analyses of the data presented in Figures S1A and S1B. Comparisons with multiple means were done using Bonferoni adjustment. The relative mRNA abundances of AARS in Met sufficiency/deprivation groups were analyzed using paired Student’s *t* test. All *P* values < 0.05 were considered statically significant. All statistical analyses were performed with SAS software (version 9.2, SAS Institute, NC, USA).

**Supplementary figures**


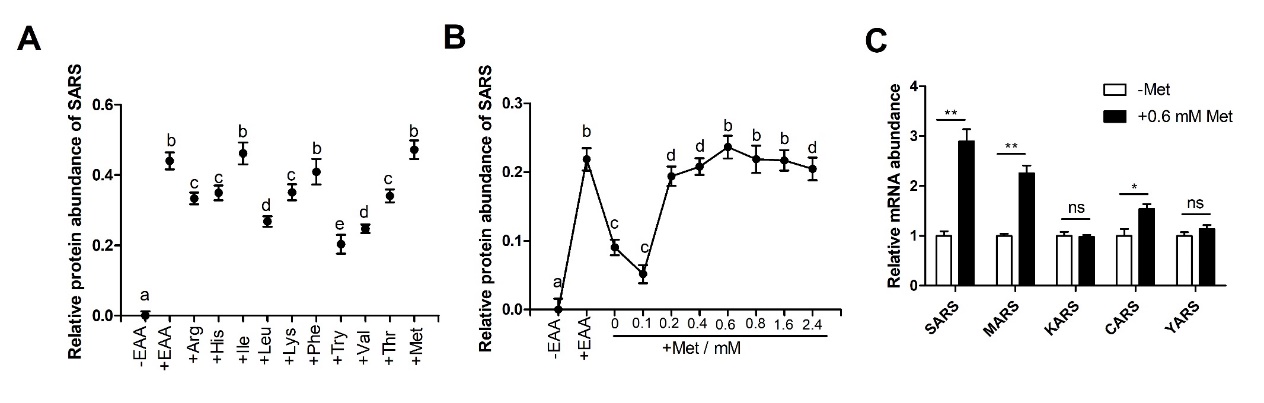


**Fig. S1** Methionine (Met) stimulates SARS (seryl-tRNA synthetase) expression in BMECs (affiliated to Figure 1). The relative protein abundance of SARS in BMECs treated with individual amino acids at the same concentrations as in the DMEM medium for 24 h (A) and different concentrations of Met (0, 0.1, 0.2, 0.4, 0.6, 0.8, 1.6 and 2.4 mM Met, respectively) for 24 h (B). +EAA indicates the DMEM medium containing all essential amino acids (EAA); -EAA indicates the DMEM medium without any essential amino acids. (C) The relative mRNA abundances of different AARS (aminoacyl-tRNA synthetases) in BMECs treated with or without 0.6 mM Met (+/-Met) for 6 h. MARS—methionyl-tRNA synthetase, KARS—lysyl-tRNA synthetase, CARS—cysteinyl-tRNA synthetase, YARS— tyrosyl-tRNA synthetase. In all panels, data represent the mean ± SEM of three independent experiments. Results in Figure S1A and Figure S1B were expressed as Log10 of the mean. The relative protein abundance of SARS in Figure S1A and S1B are analyzed using one-way ANOVA followed by Bonferoni correction, and different letters on the top indicate significant differences (*P* < 0.05). Results in Figure S1C were expressed as fold of the mean in Met-supplemented groups compared to Met-deprived groups. The relative mRNA abundance of AARS in Figure S1C is analyzed using Student’s t test; “*”: *P* < 0.05, “**”: *P* < 0.01, “ns”: no significance.

**Supplementary tables**

**Table S1** List of the primary antibodies used in Western blot assay

| Antibody | Brand | Cat No. | Production source | Dilution |
| --- | --- | --- | --- | --- |
| Rabbit-anti-SARS | Abcam | ab154825 | USA | 1:1000 |
| Rabbit-anti-S6K1 | Abcam | ab9366 | USA | 1:1000 |
| Rabbit-anti-p-mTOR^Ser2448^ | Abcam | ab84400 | USA | 1:1000 |
| Mouse-anti-β-actin | Beyotime | ab8226 | China | 1:1000 |
| Rabbit-anti-mTOR | Cell Signaling Technology | 2983S | USA | 1:1000 |
| Rabbit-anti-p-S6K1^Thr389^ | Cell Signaling Technology | 9205S | USA | 1:1000 |
| Rabbit-anti-4EBP1 | Cell Signaling Technology | 4923S | USA | 1:1000 |
| Rabbit-anti-p-4EBP1^Thr37/46^ | Cell Signaling Technology | 2855S | USA | 1:1000 |
| Mouse-anti-β-casein | Biorbyt | orb18512 | UK | 1:500 |
| Rabbit-anti-ATF4 | Bioss | bs-1531R | China | 1:500 |
| Rabbit-anti-GCN2 | Bioss | bs-2768R | China | 1:500 |
| Rabbit-anti-eIF2α | Bioss | bs-3613R | China | 1:500 |
| Rabbit-anti-p-eIF2α^Ser51^ | Bioss | bs-14541R | China | 1:500 |

**Table S2** List of RNAi sequences of SARS and MARS used in this study

| Items | Sequences of siRNA (5’-3’) | |
| --- | --- | --- |
| si-SARS | sense | GGGAGAUGUUUGAGGAGAUTT |
|  | antisense | AUCUCCUCAAACAUCUCCCTT |
| si-MARS | sense | CCGCUGCCCUGUACUAUUUTT |
|  | antisense | AAAUAGUACAGGGCAGCGGTT |
| Negative control | sense | UUCUCCGAACGUGUCACGUTT |
|  | antisense | ACGUGACACGUUCGGAGAATT |

SARS: seryl-tRNA synthetase; MARS: methionyl-tRNA synthetase.

**Table S3** List of primers used in real-time PCR of this study

| Accession | Gene | Sequences  (5’-3’) | bp |
| --- | --- | --- | --- |
| NM_001034356.2 | KARS | F:TGCAAGGCTTGTTAGGGGG;  R:AGCTCGCCCTTCTTGGTTTT. | 648 |
| NM_001103249.1 | CARS | F:AGCTGGAACCAGCCTCAATC;  R:CTCAGCTCTTTGCCTTCGGT. | 432 |
| NM_174220.2 | YARS | F:ACGATTCTGTTTGCGGACCT;  R:TTTTGGCATCGTGCTGTGTG. | 232 |
| NM_001038091.2 | MARS | F:GAGCGCTCTACCCGTTACTC; | 775 |
|  |  | R:CAGAAGGGACACACACCCTC. |  |
| NM_174175.3 | SARS | F:CCCGTGGCATCTTTAGAGTC;  R:CAGCATGATTCAAAGAACCTGAG. | 198 |
| NM_173979 | β-actin | F:GCCATGAAGCTGAAGATGAC;  R:CCTTCTGCAGCTCAGATATG. | 208 |
| DT860044 | RPS9 | F:ATGAGGGCAAGATGAAGCTG;  R:ATGAAGGACGGGATGTTCAC. | 119 |

SARS:seryl-tRNA synthetase; MARS:methionyl-tRNA synthetase; KARS:lysyl-tRNA synthetase; CARS: cysteinyl-tRNA synthetase; YARS: tyrosyl-tRNA synthetase. RPS9 and β-actin genes act as internal reference genes. The primers were designed by Primer 5.0 software (source).
